# Islet Health, Hormone Secretion, and Insulin Responsivity with Low-Carbohydrate Feeding in Diabetes

**DOI:** 10.3390/metabo10110455

**Published:** 2020-11-11

**Authors:** Cassandra A. A. Locatelli, Erin E. Mulvihill

**Affiliations:** 1 Energy Substrate Laboratory, The University of Ottawa Heart Institute, 40 Ruskin Street, H-3229A, Ottawa, ON KIY 4W7, Canada; cloca063@uottawa.ca; 2 Department of Biochemistry, Microbiology and Immunology, The University of Ottawa, Faculty of Medicine, 451 Smyth Rd, Ottawa, ON K1H 8L1, Canada; 3 Montreal Diabetes Research Centre CRCHUM-Pavillion R, 900 Saint-Denis-Room R08.414, Montreal, QC H2X 0A9, Canada; 4 Centre for Infection, Immunity and Inflammation, The University of Ottawa, 451 Smyth Rd, Ottawa, ON K1H 8M5, Canada

**Keywords:** low carbohydrate diet, ketogenic diet, insulin sensitivity, pancreas, islet of Langerhans

## Abstract

Exploring new avenues to control daily fluctuations in glycemia has been a central theme for diabetes research since the Diabetes Control and Complications Trial (DCCT). Carbohydrate restriction has re-emerged as a means to control type 2 diabetes mellitus (T2DM), becoming increasingly popular and supported by national diabetes associations in Canada, Australia, the USA, and Europe. This approval comes from many positive outcomes on HbA1c in human studies; yet mechanisms underlying their success have not been fully elucidated. In this review, we discuss the preclinical and clinical studies investigating the role of carbohydrate restriction and physiological elevations in ketone bodies directly on pancreatic islet health, islet hormone secretion, and insulin sensitivity. Included studies have clearly outlined diet compositions, including a diet with 30% or less of calories from carbohydrates.

## 1. Introduction

Diabetes mellitus (DM) is a life-altering condition affecting a rapidly growing population worldwide. Type 2 (T2) DM accounts for the majority of this increase and is characterized by hyperglycemia resulting from insufficient endogenous insulin secretion due to beta cell dysfunction or hyperinsulinemia and impaired tissue response to insulin [1]. Appropriately integrated hormonal signals are paramount to initiating proper fasted and post-prandial responses to control glycemia. Both glucose and active incretin peptides stimulate membrane depolarization of pancreatic beta cells and subsequent insulin secretion [2,3]; however, overstimulation of beta cells can lead to impaired secretory function and apoptosis [4]. Thus, modulation of dietary carbohydrate has long been considered for the treatment of DM. Notably, extreme carbohydrate restriction was commonly advised to patients with diabetes before the discovery of insulin [5]. However, recommendations by leading national diabetes organizations have not remained consistent with this initial paradigm, with fat restriction being commonly recommended from the 1970s until recently [6,7]. Since 2017, Diabetes Canada, Diabetes UK, Diabetes Australia, and the American Diabetes Association have approved the use of low-carbohydrate diets (LCD) and ketogenic diets (KD) with support from a physician to manage T2DM [8,9].

Restriction of dietary carbohydrates has become increasingly popular, specifically in T2DM, to improve glycemic control. Increasing the time spent in an optimum glucose range (~4–7 mM) [10] is an important clinical target since the Diabetes Control and Complications Trial that demonstrated that even small deviations in glycemia throughout the day significantly increases risks of secondary diabetic complications [11]. It is understood that beta cells are sensitive to apoptotic signals and oxidative stress in conditions which are exacerbated by overnutrition and obesity [12,13]. Insensitivity to insulin by other metabolic tissues can promote hyperinsulinemia, beta cell hyperplasia, and contribute to beta cell failure. Additionally, hepatic insulin resistance also contributes to hyperglycemia by failing to suppress glucose production [14,15]. Insulin insensitivity, lipid accumulation in the pancreas, and elevated circulating insulin concentrations have been reproducibly demonstrated as outcomes of a Western-style obesogenic diet in animal models [16,17,18,19]. T2DM is a heterogeneous condition; however, particularly in the early stages, it is postulated that an LCD may reduce metabolic strain, oxidative stress, and proinflammatory conditions to positively impact beta cell health. However, this has been incompletely demonstrated.

The KD is a very-LCD, typically less than 10% of kcal from carbohydrates, which can induce the fasted-like state of ketosis. During ketosis, excess acetyl-CoA from the catabolism of lipids by hepatic beta-oxidation can be used for ketogenesis, the production of ketone bodies acetoacetate, beta-hydroxybutyrate (BHB), and acetone. Extra-hepatic tissues can take up ketone bodies for ketolysis and generation of ATP through the tricarboxylic acid cycle and electron transport chain [20]. This diet has been historically used to treat children with intractable epilepsy [21]. Further, supplementing exogenous ketones while consuming other diets has also been explored for potential metabolic benefits [22]. These studies provide insight into the effects of ketone bodies independent of the reduced insulin requirement by carbohydrate restriction of KD. It is currently unclear whether the ability of a diet to achieve or maintain ketosis plays a large role in the improved glucose regulation seen with KDs in patients living with DM.

LCD and KDs have shown clinical benefit through reduced need for DM medication in many patients with T2DM and have even been recommended by clinicians as a first approach [23]. However, large-scale, well-controlled trials in patients with T2DM are limited and preclinical research to determine the mechanisms of observed improvements in human trials remains largely controversial. Further, these studies are confounded by the effects of weight loss, appetite suppression, and differing dietary composition of the intervention [24,25].

The aim of this review is to discuss the effects of LCDs, KDs, and dietary supplementation with exogenous ketones on DM as it pertains to: (1) physiology of insulin-secreting beta cells, (2) secretion of islet hormones insulin and glucagon, and (3) sensitivity of other tissues to insulin.

For the purposes of this review, LCDs are defined as having less than 30% of calories from carbohydrates and diets referred to as KD have shown evidence of ketosis through significantly elevated circulating ketone bodies (See Table 1 for all diet definitions). These definitions were used for consistency between rodent, non-human primate, and human studies. Studies included had clearly outlined diet compositions and used LCDs and KDs that are calorically unrestricted, isocaloric to previous eating patterns, or without intentional caloric restriction past control diets. Pubmed search terms included “low carbohydrate islet”, “ketogenic diet islet” “low carbohydrate diabetes” “ketogenic diet diabetes”. The role of ketones in cancer, including pancreatic cancer have been reviewed elsewhere [26,27].

## 2. Islet Health and Survival

Progressive beta cell dysfunction and beta cell death are key features of T2DM. Beta cells compensate for insulin insensitivity through hyperplasia and related hyperinsulinemia before a dramatic loss of beta cells. This leads to insufficient insulin production, impaired glucose tolerance, and exogenous insulin dependence [28,29,30]. Both ectopic lipid accumulation and hyperglycemia induced by Western diet feeding have been demonstrated to promote beta cell toxicity and death in rodents [16,31]. Whether the same pathways are engaged in patients is a matter of debate [32]. Although many KD and LCD studies focus on weight loss and glycemia, histological analysis of pancreatic islet composition in animal models provides important insight into the health and survival of insulin and glucagon secreting beta and alpha cells which remain unknown in human trials. These studies may provide insight into the mechanisms behind the need for altered insulin or anti-hyperglycemic medication which is characteristic of patients with diabetes in trials of LCDs.

### 2.1. Non-Obese, Non-Diabetic Animal Studies

**Figure 1:**
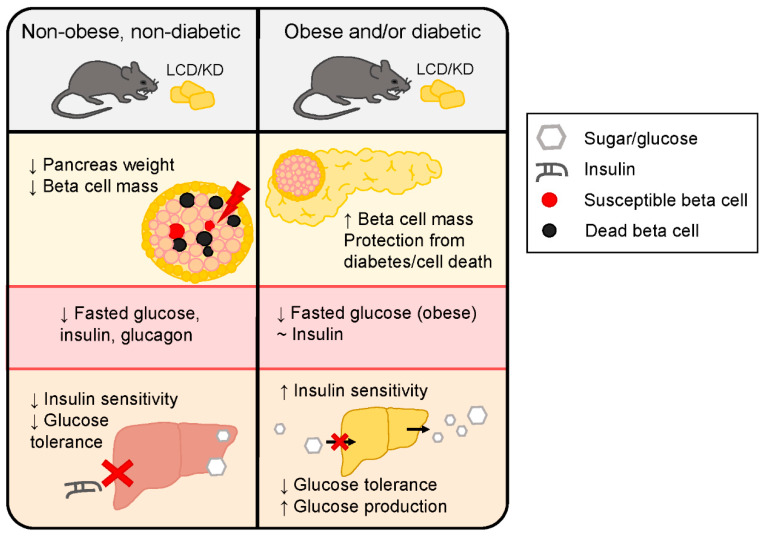
Extreme carbohydrate-restricted diets may confer some benefits in obese and diabetic rodents compared to healthy rodents. Some reports of ketogenic diet (KD) and carbohydrate-free diets (CFDs) demonstrate protection against the onset of diabetes with improved beta cell survival or proliferation after streptozotocin treatment. Low-carbohydrate diets (LCDs) improve fasted and random glycemia but worsen insulin responsivity during hyperinsulinemic euglycemia clamp studies and increase glycemia during glucose tolerance tests.

Interestingly, few positive impacts have been reported on pancreatic islet health in rodents fed LCD and KDs in studies using non-obese, non-diabetic models (Figure 1). C57BL/6 mice fed a KD for 22 weeks showed a greater than 50% decrease in alpha and a ~30% decrease in beta cell mass compared to chow-fed mice without changes to islet density, suggesting increased size of the remaining cells [33]. Similarly, male Wistar rats fed LCD or KD for 4 weeks had decreased pancreas and beta cell volume compared to chow-fed rats, even when normalized for body weight [34]. A recent study of young male and female C57BL/6 mice on three different LCDs for 12 weeks found that only mice on the 1% carbohydrate LCD (0 kcal sucrose) had similar beta cell proliferation (% Ki-67 positive cells) rate as low-fat diet controls. In males, both 20% carbohydrate LCDs had increased proliferation, associated with hyperplasia, whereas only the 20% carbohydrate diet with higher sucrose (775 kcal vs. 275 kcal) was significantly elevated in females, despite identical macronutrients. There were no differences in beta cell mass between diet groups in females, but male mice on the 1% carbohydrate LCD had significantly reduced beta cell mass than even low-fat controls, despite similar proliferation rate [35]. In contrast, studies using obese and/or diabetic models did not report differences between non-obese, non-diabetic control diet groups [36,37,38].

### 2.2. Animal Models of Diabetes and/or Obesity

In two studies by the same group, rats with streptozotocin-induced diabetes consuming 10% carbohydrate LCDs had more beta cells than rats fed a high-carbohydrate diet. In the study with a higher fat and lower protein LCD (60% fat, 30% protein), rats had less beta cells than chow-fed controls, suggesting that this diet was inferior for islet mass preservation compared to typical healthy feeding. [36,37]. More strikingly, streptozotocin-treated rats fed the very high protein LCD (60% protein 30% fat) did not show signs of streptozotocin-induced beta cell death and islet morphology perturbations, unlike in both chow and high-carbohydrate-fed rats [36]. Notably, diabetes was induced by streptozotocin at different times in these two studies. The higher fat LCD experiment induced diabetes at the onset of dietary intervention whereas in the experiment with higher protein LCD, dietary intervention began 8 weeks prior to streptozotocin treatment. In both studies, chow and high carbohydrate groups increased their food intake after streptozotocin treatment, but LCD-fed rats did not [36,37].

In the diabetic susceptible mouse model *db/db*, Mirhashemi and colleagues found that a carbohydrate-free diet (CFD) preserved beta cells and expression of glucose transporter (GLUT2) after 22 weeks of dietary intervention. Both the standard chow and obesogenic HFD feeding resulted in diminished beta cell count [39]. Similarly, a 1983 study on the role of carbohydrate in *db/db* mice found that the CFD-fed mice showed some islet hyperplasia and hypertrophy, but significantly reduced islet atrophy and improved survival compared to those on diets of varied carbohydrates (8–60%) [40]. These results are corroborated in New Zealand Obese (NZO) mice fed a CFD for 22 weeks [41], but not NZO mice fed a very LCD (6% kcal from sucrose) for 9 weeks; here, Lamont et al. found no changes to pancreas weight, islet density, islet size, or beta cell mass [42]. These results suggest a benefit on islet health in rodents with a total lack of carbohydrate, not only carbohydrate restriction, as higher sucrose (compared to 2% in standard diet) is detrimental to beta cell health [40]. However, this raises questions on the durability of these effects upon the reintroduction of carbohydrates after a CFD. After 18 weeks on a CFD, Kluth et al. fed NZO mice a diet with 32% of kcal from carbohydrates or continued the CFD. When refed carbohydrates, mice quickly developed diabetes demonstrated by loss of insulin-positive cells and increased caspase 3 expression [43]. It is important to note that the carbohydrate refeeding diet was proportionately closer to a typical HFD (51.4% of kcal from fat, 32.4% kcal from carbohydrate), which is known to be metabolically deleterious. However, a study on chow refeeding in rats after 8 weeks of KD demonstrated hyperphagia and enhanced weight gain compared to mice consistently fed a chow diet. Islet and pancreas analyses were not performed [44]. Finally, in the *ob/ob* genetic model of obesity, mice fed an LCD had reduced beta cell mass compared to *ob/ob* chow-fed mice, similar to levels in wildtype chow fed mice, indicating hyperplasia in the chow-fed *ob/ob* mice which is attenuated by the LCD [38].

## 3. Islet Hormone Secretion

Constant glucose homeostasis as a result of appropriate secretion of metabolism-regulating islet hormones or treatment with diabetes medication is important for daily metabolism, avoidance of hyper- and hypoglycemic events, and is crucial for patients with diabetes to avoid long term complications [11]. Physiological regulation of glucose by the endocrine pancreas occurs through the integration of nutrient signals with a series of electrical gradients and molecular processes which result in the secretion of glucoregulatory hormones, insulin and glucagon, from the islets of Langerhans. Investigating the effects of LCD, KD, and their metabolites on glycemic load and islet secretory capacity compared to standard diets is paramount to understanding their role in DM and its comorbidities.

### 3.1. Cell Studies

**Figure 2:**
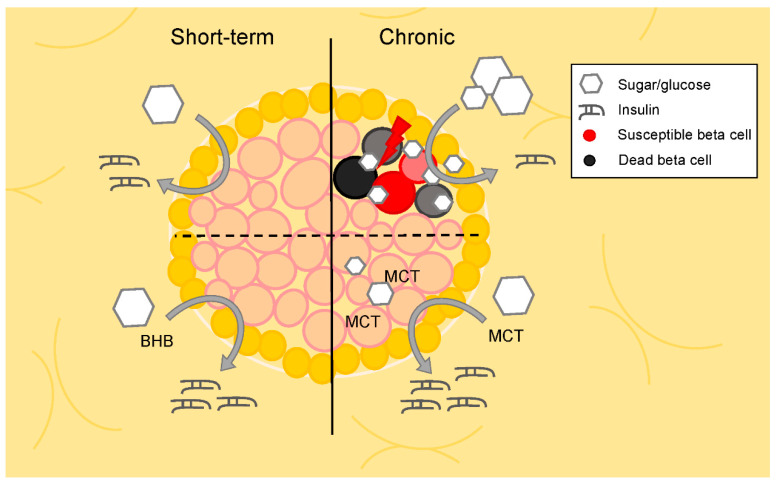
Short-term and chronic effects of metabolites on pancreatic beta cell insulin secretion. In vitro investigations in isolated islets reveal an additive effect of BHB and glucose on insulin secretion, but no insulin secretion with BHB alone. While long-term treatment with high glucose can lead to reduced glucose-stimulated insulin secretion, some evidence suggests increased insulin secretion in response to high glucose following chronic (72 h) treatment with ketones and medium chain triglycerides. BHB: beta-hydroxy butyrate; MCT: medium chain triglycerides.

The canonical mechanism of insulin secretion occurs in response to glucose; however, in vitro studies in isolated rat and human islets have shown that exposure to BHB in addition to glucose further increases insulin secretion but does not have a significant effect alone [45,46,47,48,49]. Further, while acetoacetate, BHB, and monomethyl succinate had no significant effects on insulin secretion in INS-1 cells alone, incubation of either acetoacetate or BHB with monomethyl succinate induced significant insulin secretion [48]. Similarly, monomethyl succinate and BHB together also stimulated insulin secretion in isolated rat islets, though neither alone [48]. Interestingly, one 1995 study shows that treatment of human islets for 48 h with BHB at concentrations seen in patients with uncontrolled type 1 DM impairs insulin secretion in the presence of high glucose media [50]. Conversely, a recent study found that supplementing high-glucose media with medium chain triglycerides improved glucose-stimulated insulin secretion in isolated beta cells from aged rats [51]. Chromic (72 h) treatment with medium chain triglycerides or BHB also increased glucose-stimulated insulin secretion in the INS1E beta cell line [51] (Figure 2). Proinsulin biosynthesis in islets from *ob/ob* mice, in significant contrast to glucose, was unchanged in the presence of ketone bodies [52]. A number of studies have evaluated the expression and activity of ketone body transport and utilization enzymes within islet cell lines, primary pancreatic tissue [53,54] and within mouse islets [55]. Additionally, oxidation rates in islets isolated from *ob/ob* mice (6–8 months old) determined conversion of ketone bodies to CO_2_ occurs at significant rates albeit much lower than other tissues, including the kidney [56]. However, surprisingly few studies have explored the physiological adaptation of islets to carbohydrate restriction, nutritional ketosis, or consumption of ketone esters on the dynamics of hormonal secretion and metabolism within islet cell populations.

### 3.2. Healthy Animal Models

In non-obese, non-diabetic rodent studies, LCD and KD feeding often results in significantly lower fasted glucose than rodents on a chow diet [34,57,58,59,60], or no different than chow or low-fat diet [33,35,61,62] and improved compared to obesogenic HFDs. Fed state glucose was also decreased in KD compared to chow-fed mice [61]; however, it was not different than chow-fed rats [37,63]. When challenged with systemic (insulin tolerance test) or neural hypoglycemia, mice on the KD for 7 days had significantly lower glucagon and blood glucose compared to chow-fed mice, indicating reduced protection for hypoglycemic events [60]. Jornayvaz et al. similarly found that mice fed a KD had decreased fasted glucagon compared to chow-fed mice [59], whereas in another study, KD mice had similar fed glucagon to weight matched, chow-fed controls [61]. Fasted insulin has been reported to decrease with KD feeding compared to chow feeding [34,57,59,61,62,64]; however, other studies report that fasted insulin is not different between KD and chow-fed mice, although decreased compared to HFD-fed male mice [35,58]. Interestingly, in the longest running study we evaluated, mice fed a KD for 22 weeks had increased fasted insulin compared to chow-fed mice [33]. Finally, non-human primates fed standard high carbohydrate diet had less insulin and glucose perturbations with a LCD meal compared to high-carbohydrate, low-fat meal [65].

### 3.3. Animal Models of Obesity and Diabetes

Unlike in healthy rodents, KD feeding in rodent models of obesity and diabetes have more varied fasted glucose outcomes. In *ob/ob* and diet-induced obese mice, fasted glucose was lower in KD-fed mice than in chow-fed mice [38,57,61]. However, fasting glucose was not significantly different from chow-fed rodents in pancreatectomized, obese, or streptozotocin treated rodents fed LCD and KD [63,66,67] and was increased in LCD-fed NZO mice [42]. Unsurprisingly, fed glucose levels in obese and diabetic rodents on LCDs are consistently lower than chow-fed rodents [37,38,42,61,68]. Interestingly, Park et al. report pancreatectomized KD mice had more than doubled average fasted glucagon levels than pancreatectomized chow and exogenous ketone-supplemented groups [66], whereas Badman et al. found decreased fed glucagon levels in KD compared to chow-fed *ob/ob* mice [61].

Unlike in the healthy rodents on LCDs who reproducibly have lower fasted insulin, only one study using a mouse model of obesity (*ob/ob*), had decreased fasted insulin as compared to chow fed mice [61]. Others, using pancreatectomized rats, streptozotocin treated rats, or obese (NZO) mice, showed no change in fasted plasma insulin levels with LCD, KD, or exogenous ketones compared to chow [42,66,67]. Using a CFD, Mirhashemi et al. found that non-fasted insulin was consistent over time in *db/db* obese mice despite a decrease in insulin in HFD-fed mice at 6 weeks and chow-fed mice by the endpoint (22 weeks), suggesting decompensation [39]. Finally, non-fasted insulin levels of obese LCD and KD rodents tended to be decreased compared to higher carbohydrate chow and HFDs [38,57,61,63,68]. Although, one study using NZO mice found increased fed insulin levels after 9 weeks of LCD feeding [42]. In all, LCD and KDs protect rodents from hyperglycemia and large glycemic fluctuations.

### 3.4. Human Studies

In most cases, human studies on LCD and KD feeding report significantly reduced fasted and 24 h insulin and increased fasted, non-fasted, and 24 h glucagon compared to control groups or baseline measurement [69,70,71,72,73,74]. Interestingly, Samaha and colleagues found decreased insulin levels in severely obese participants currently without DM medications, but not with those using DM medications [75]. In contrast, a 52-week study comparing LCD and high carbohydrate hypocaloric diet found no difference in serum insulin between groups [76]. Similarly, a 2-week crossover study found no changes to insulin or glucagon at basal insulin before hyperinsulinemic clamp [77].

Measurement of HbA1c provides a much more complete picture of glycemia than one-time fasted or fed glucose measurement and is indicative of inadequate insulin secretion or glucose uptake over time. Impressively, studies in patients with T2DM on LCD and KD both randomized and non-randomized, controlled and uncontrolled trials, from 2 weeks to 44 months reproducibly resulted in decreased HbA1c [69,71,72,75,78,79,80,81,82,83,84,85,86]. Similarly, case studies of varying lengths tended to also find improvements in HbA1c after 20 weeks [87]. Controlled studies have found this improvement in HbA1c on LCD and KDs compared against diets with the majority of energy coming from carbohydrates, diets with low glycemic index and calorie restriction, and caloric restriction alone [69,79,81,84]. In addition, continuous care regimens using KDs have be shown to greatly improve HbA1c in patients with T2DM compared to current standard T2DM care [88,89]. Strikingly, many participants in the continuous care group with KD had reversal (53.5%) or remission (17.6%) of their T2DM [88]. While continuous care interventions introduce the confounding variables of extra support and resources, these studies offer insight into the potential for improved care in conjunction with the benefits demonstrated by other trials of CRDs alone. Conversely, one 6-month study comparing a LCD and a low-fat Diabetes UK recommended diet, HbA1c only trended towards improvement in the LCD compared to the control diet among participants with DM (*p* = 0.06) [75]. This study saw significantly decreased weight compared to the low-fat group, and the decrease in fasting glucose that was seen in diabetic patients was no longer significant after controlling for weight loss. In a 24 weeks study, Dyson and colleagues found HbA1c was only significantly decreased with LCD feeding compared to baseline and not the control low-fat diet when both patients with and without DM are considered and not DM alone, despite increased weight loss with LCD compared to the low-fat diet [25]. Similarly, a 52-week study found that improvements in HbA1c were not different than those of the high carbohydrate group [76]. Remarkably, Yancy et al. found that the decrease in HbA1c was greater than that which is predicted by the weight loss and Vernon et al. found that HbA1c improvement was observed in patients with and without weight loss, alike [78,80]. Additionally, by nature of carbohydrate restriction and the positive outcomes above, human studies on the CRDs almost always result in decreased reliance on anti-hyperglycemic agents and/or lowering of insulin dosage [69,71,75,76,77,79,80,81,83,84,90], and on occasion even significantly more than calorie-restricted and low glycemic index diets [69,79].

## 4. Insulin Sensitivity

Insulin insensitivity is an important hallmark of T2DM as a lack of tissue responsiveness to insulin remains a barrier to stabilizing glycemia. In rodent studies, there are a number of accepted methods for testing whole body and tissue-specific insulin sensitivity. The gold standard for insulin sensitivity measurement is the hyperinsulinemic-euglycemic clamp; however, glucose and insulin tolerance tests are very widely used. Additionally, when dynamic physiological tests are not performed, simple blood draws can be used to measure homeostatic model assessment for insulin resistance (HOMA-IR) and quantitative insulin sensitivity check index (QUICKI). While studies demonstrate consistencies between methods in humans, such comparisons between methods in rodents are not as appropriate [91,92,93].

### 4.1. Non-Obese, Non-Diabetic Mouse Models

In both C57BL6 mice fed a KD for 3 days and 5 weeks and Wistar rats fed LCD and KD for 3 weeks, hyperinsulinemic-euglycemic clamp studies show reduced hepatic insulin sensitivity and inability to suppress endogenous glucose production [34,58,59]. Additionally, in two of those studies, rodents had impaired uptake of glucose during the clamp experiment [34], specifically in heart and brown adipose tissue [59]. Most insulin tolerance tests (ITT) on healthy mice fed CRDs report lower or the same glycemia compared to a standard chow diet [33,60,62,94]. Notably, two KD-fed mice in one study became hypoglycemic during the ITT and required rescue with dextrose solution [62]. However, the shortest study included, with only 3 days on KD, reported increased area under the curve of glucose compared to both a chow and obesogenic HFD during the ITT [58]. With only three days on the diet it is possible that this represents a metabolic transition phase rather than an established state [58]. In non-obese, non-diabetic rats, three studies found that both LCD and KD impaired insulin tolerance through elevated glucose during the ITT compared to chow-fed rats [34,63,64]. Similarly, Kinzig and colleagues found that feeding a liquid high carbohydrate meal to rats on the LCD significantly increased circulating insulin and glucose for 2 h after feeding compared to chow-fed rats [64]. Non-validated measurements of insulin sensitivity in rodents, QUICKI and HOMA-IR, were improved in studies of LCD and KD in healthy wildtype mice [59,62]. Studies in healthy rodents agree that CRDs impair glucose tolerance as assessed by oral and intraperitoneal glucose tolerance test [33,34,35,38,58,62,64,94,95]. Interestingly, a recent study revealed that in healthy mice on diets with fixed 25% protein, those with fat content of 41.7% and above were all glucose intolerant; this phenomenon was not seen with 10% fixed protein, despite that being much closer to a typical KD [96].

### 4.2. Mouse Models of Obesity and Diabetes

Similar to healthy rodents, rodent models of diabetes and obesity fed CRDs had poorer outcomes during hyperinsulinemic-euglycemic clamp experiments than chow-fed controls. NZO mice fed a CFD fared similarly to HFD-fed mice with decreased glucose infusion rate to achieve euglycemia, decreased glucose utilization, and decreased suppression of hepatic glucose production compared to chow-fed mice [41]. Pancreatectomized rats on KD also had lower glucose infusion to achieve euglycemia and increased hepatic glucose output. No differences were found in glucose uptake between KD, chow, and BHB injection groups [66]. Interestingly, unlike KD rats, rats administered exogenous BHB had decreased hepatic glucose output compared those on chow [66]. During hyperglycemic clamp, BHB injected and KD rats had reduced first phase insulin secretion while only BHB injected rats had reduced second phase insulin [66]. Both KD and BHB groups had reduced glucose infusion rate and insulin sensitivity, measured as milligrams of glucose per kg of body weight over time [66]. This speaks to a potential benefit of increased fat content of KD over exogenous BHB which may allow for adequate second phase insulin secretion. During ITTs, obese (*ob/ob*) mice had lower blood glucose than chow-fed controls, indicating better insulin sensitivity; however, these mice again were glucose intolerant. Mice with diet-induced obesity switched to an LCD were also glucose intolerant but they had similar insulin tolerance to those switched to chow [35]. Similarly, LCD-fed rats treated with streptozotocin had similar ITT response as non-streptozotocin chow fed rats, but their glucose tolerance, while improved at some time points, did not have significantly improved area under the curve (AUC) glucose compared to HFD streptozotocin controls [67]. Genetically obese (*fa/fa*) rats fed an LCD with high protein (34.7% kcal) also had similar insulin tolerance to chow-fed controls, with increased AUC of glucose and insulin during a glucose tolerance test [63]. Conversely, two studies found that KD-fed mice had similar glucose tolerance to chow-fed mice and improved glucose tolerance compared to HFD-fed mice in models of diet and genetically (*ob/ob*) induced obesity [57,61]. Insulin sensitivity, as determined by QUICKI, was improved in obese (*ob/ob*) mice [61].

### 4.3. Human Studies

**Figure 3:**
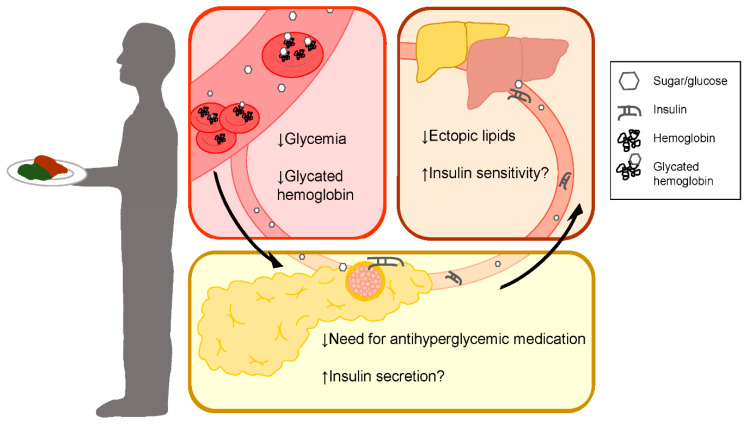
Low-carbohydrate diets reduce the need for anti-hyperglycemic agents in type 2 diabetes. Participants on LCDs with T2D have decreased glycemia, glycated hemoglobin, and improved blood glucose regulation; thus, require less exogenous insulin and other anti-hyperglycemic medications and may improve insulin sensitivity through restored insulin secretion in some patients.

Two human studies have assessed insulin sensitivity using hyperinsulinemic-euglycemic clamps in LCD and KD fed participants with T2DM. In contrast to rodent studies, neither short term, small population study had worsened insulin sensitivity with LCD and KD [71,77]. Notably, KD fed participants in one study required significantly more glucose to maintain euglycemia than the same participants did on their prior diets [71] (Figure 3). This inpatient study lacked a control group; therefore, these improvements may be a reflection on the poor diet they consumed prior to the study but may also serve as a more realistic comparison for the average person with T2DM eating a Western-style diet [71]. Allick et al. compared their findings against a eucaloric, high carbohydrate diet and found no differences between groups during the clamp study despite significantly worsened hepatic insulin sensitivity in a similar study done on non-diabetic participants [77,97]. Additionally, in a study of non-diabetic, obese adults over 60, participants on the LCD for 8 weeks had improved insulin sensitivity during hyperinsulinemic-euglycemic clamp compared to baseline, while the low-fat group saw no significant change. The difference between diet groups was not significant [73]. In mixed meal tests, Rosenbaum et al. found that 17 healthy participants fed KD versus a baseline 50% carbohydrate diet had worse insulin sensitivity with elevated AUC glucose during ketogenic and carbohydrate-based meals and elevated AUC insulin during the carbohydrate-based meals [74]. Further investigation is required to assess insulin sensitivity on LCDs in the context of T2DM.

## 5. Paracrine Regulation and Metabolic Crosstalk

Paracrine regulation of islet hormone secretion may also be of importance to the LCD and diabetes literature. The incretins glucagon-like peptide 1 (GLP-1) and glucose-dependent insulinotropic polypeptide (GIP) are secreted by L and K cells in the intestine in response to nutrients [2]. Secreted, active GLP-1 and GIP can stimulate beta cells of the pancreas to potentiate glucose-stimulated insulin secretion, with GLP-1 having the added effect of inhibiting glucagon secretion from alpha cells [2]. However, the incretins are quickly cleaved and inactivated by dipeptidyl peptidase-4 (DPP4) and cleared through the kidney [98]. Both DPP4 inhibitors and GLP-1 receptor agonists are currently in use as DM medications [99,100]. One study reported that normal weight participants taking a ketone ester drink had significantly lower total GLP-1 two to four hours after drink administration compared to those taking a dextrose drink [101]. Similarly, Wallenius and colleagues reported that ketone bodies reduce GLP-1 secretion in primary jejunal culture [102]. Alternatively, a small, uncontrolled study reported increased fasted GIP in healthy patients on a KD but no changes to GLP-1 [74]. The differences here may be due to the vastly different diets; very low-calorie diets and ketone ester drinks do not provide the extreme fat content of a calorically unrestricted KD. Dietary fatty acids can signal through G protein coupled receptors in the intestine which can potentiate GLP-1 and GIP secretion [103,104,105]. Further studies are required to understand the relationship between incretin secretion and KD for any therapeutic benefits.

## 6. Discussion and Conclusions

In summary, the preclinical literature supports the concept that islet health may be positively impacted by CRD in states of metabolic disease rather than in the healthy state. Much of the literature on dietary modulation on pancreas and islet health centers on beta cell destruction; as such, the diet regime is extreme with many studies using diets free of carbohydrates, which clearly limits its translational value. Understanding the functional impact of different dietary regimes on the energy sources presented to the beta cell becomes increasingly relevant as we learn more on the importance of lipid storage within the pancreas, integrity of mitochondria and oxidative metabolism on insulin secretion and the development of diabetes [106,107]. Additionally, it is clear, given the significant effects of diets with a high percentage of fat reported on body weight, food intake, and energy expenditure [108], that metabolic cross-talk from insulin sensitive tissues including adipose tissue, liver, and skeletal muscle may also very dramatically influence the islet and metabolic outcomes associated with the feeding of LCD or KD. CRDs with differing composition should be studied more closely and rigorously to address the mechanisms underlying reported inconsistencies and the translational value of these studies. Additionally, exercise can play an important role in the management of diabetes and other metabolic dysfunction [109,110,111]. There is some evidence to suggest that an LCD or KD may impair exercise ability [112,113,114,115], although this is not consensus and may depend on exercise type [116,117,118]. Further studies on the role of ketosis and carbohydrate restriction on exercise capacity in patients with diabetes is warranted.

Carbohydrate restriction (<30% kcal) has demonstrated benefits in human studies of T2DM with regulating glycemia and reducing the need for DM medications. Interventional studies in patients with T2DM examining changes in glycemia, insulin sensitivity, and beta cell function provide valuable insight into the mechanisms underlying the consistency by which LCD or KD may improve HbA1c levels. The value of improved glycemic control is evident given the significant role of the length of exposure to hyperglycemia in the development of complications. It is currently unclear how factors which influence the heterogeneity of T2DM including age, race, and genetics [119] influence the ability of carbohydrate restriction to resolve morbidity. In addition, there is substantial debate about the heterogeneity in the sources of fat present in the diet [120,121,122] which may also influence the consistency of these outcomes. Further, like any dietary restriction or modulation, adherence is a major obstacle to overcome when informing clinical practice and evaluating long-term outcomes. While carbohydrate consumption was decreased in a study aiming to explore an LCD, participants randomized to the LCD group did not have significantly increased fat intake compared to controls, rather decreased caloric intake [123]. A 2018 review also highlights the difficulty of adhering to a KD [124]; thus, studying the transition from a more restrictive KD to an LCD for the long term may be more clinically relevant.

## Figures and Tables

**Table 1:** Dietary definitions used for the purpose of this review. Percentages are percent of kilocalories.

Diet			Carbohydrate	Fat	Protein
**CRD**	Carbohydrate-restricted diet	A diet which intends to decrease carbohydrate consumption	<40%	>30%	4–60%
**LCD**	Low-carbohydrate diet	A CRD with less than 30% of kcal from carbohydrates without evidence of elevated ketone bodies	<30%	30–95%	4–60%
**KD**	Ketogenic diet	An LCD with elevated ketone bodies but some dietary carbohydrate and typically low protein	<10%	>70%	4–20%
**CFD**	Carbohydrate-free diet	A diet containing no carbohydrates (preclinical)	0%	8–88%	12–83%
